# Dihydroquercetin in Obesity and Prediabetes: Case Report and Insights from Molecular Modeling

**DOI:** 10.3390/ijms27062846

**Published:** 2026-03-20

**Authors:** Roman P. Terekhov, Amir Taldaev, Artem A. Svotin, Denis I. Pankov, Evgenia M. Sukhova, David A. Manukov, Ketelina Bergel, Maria D. Korochkina, Irina A. Selivanova

**Affiliations:** 1 Nelyubin Institute of Pharmacy, Sechenov First Moscow State Medical University, 119991 Moscow, Russia; 2 Laboratory for the Study of Single Biomacromolecules, Institute of Biomedical Chemistry, 119121 Moscow, Russia; 3 Research Center for Molecular Mechanisms of Aging and Age-Related Diseases, Moscow Center for Advanced Studies, 123592 Moscow, Russia

**Keywords:** obesity, prediabetes, dihydroquercetin, flavonoid, clinical case, molecular docking, molecular dynamics

## Abstract

Dihydroquercetin (DHQ) is a promising object for the development of a treatment for patients with obesity and prediabetes requiring a moderate therapeutic effect. This paper reports a clinical case of DHQ application in a 30-year-old Caucasian male and proposes a molecular mechanism of its anti-obesity effect. DHQ was administrated as a dietary supplement at a dose of 100–200 mg/day during 3 months with treatment interruption for 1 month. The data collected one month before the treatment were used as a control. The molecular aspects were studied via molecular docking with β_3_-adrenoceptor (ADRB3, PDB ID: 9IJE) and peroxisome proliferator-activated receptor γ (PPARG, PDB ID: 2ZNO) and molecular dynamic simulation under conditions mimicking a human cellular environment. A pronounced weight decrease up to 0.73 kg/week was observed during DHQ administration. The highest affinity to ADRB3 was observed for the non-ionized H2*a*H3*e*-conformation of 2*S*,3*R*-DHQ (–8.846 kcal/mol). Molecules with 2*S*-configuration demonstrate 0.332 kcal/mol higher affinity to PPARG compared to 2*R*-stereoisomers. The intermolecular complex with *cis*-DHQ demonstrated higher stability in molecular dynamics simulation. The insights gained from this study may contribute to our understanding of flavonoids not merely as antioxidants but also as active ingredients that selectively interact with receptors. If future investigations confirm these results, they may serve as a foundation for developing a new class of anti-obesity remedies that act via ADRB3.

## 1. Introduction

Obesity is a problem that was classified by the World Health Organization as a global epidemic [1]. A body mass index (BMI) higher than 30 kg/m^2^ is categorized as class I of obesity. The prevalence of overweight and obesity is increasing, with an estimated 3 billion adults expected to be overweight or obese by 2030, up from 1.6 billion in 2010 [2]. Frequently, such patients’ medical histories are burdened by diabetes mellites and other metabolomic disorders that also need to be treated [3,4,5]. Over the last few years, glucagon-like peptide-1 receptor agonists (GLP-1RAs) have become the mainstream treatment for such patients [6]. However, there is a need for the development of medications for obesity treatment tailored to the individual characteristics of patient subgroups.

Natural compounds with anti-obesity activity may be a treatment of choice for patients requiring a moderate therapeutic effect [7]. For example, dihydroquercetin (DHQ), also known as taxifolin, is a promising object for development [8]. Larch wood is used for the industrial production of this bioflavonoid [9]. Additionally, DHQ was found in rice [10], ziziphus jujube seeds [11], and Korean rhododendron [12], among other plants. In recent years, biotechnological approaches for flavanonol synthesis are being actively explored [13,14,15].

DHQ is known for its wide range of biological activity, including prevention of age-related dementia [16] and diabetes [17], accelerating of wound healing [18,19], and others. However, the molecular mechanisms of these multiple effects are not clear yet. The application of molecular modeling approaches in cooperation with clinical data may shed a light on this question.

This paper reports a clinical case of DHQ application in an obese patient and proposes a molecular mechanism of its anti-obesity effect, supported by molecular docking and molecular dynamics simulations.

## 2. Case Presentation

### 2.1. Clinical Observation

We describe the case of a 30-year-old Caucasian male who was diagnosed with class I obesity (BMI = 32.1 kg/m^2^) and prediabetes (fasting blood sugar, FBS = 6.1 mmol/L) in August 2025. The complaints included weight gain over the past 6 months, walking dyspnea, and hypertension. The patient is a white-collar worker with a sedentary lifestyle. The calculated dietary energy intake in the last days before the visit was about 2900 kcal/day, while the dietary energy requirement has to be 2560 kcal/day. There is no history of acute myocardial infarction, cerebrovascular accidents, or erectile dysfunction. Physical examination did not detect any skin striae or hyperpigmentation. Height was 1.78 m, and weight was 101.7 kg. Blood pressure and heart rate were 142/86 mmHg and 98 BPM, respectively. Biochemical analysis showed no evidence of testicular dysfunction.

In accordance with the clinical guidelines, a change in lifestyle was recommended as the main treatment. It included a diet with a calorie deficit (2000 kcal/day), avoidance of fasting, and moderate physical activity (with a heart rate 110–140 BPM) for at least 150 min/week. To comply with the principles of balanced nutrition, the Harvard Healthy Eating Plate guideline was suggested [20]. To evaluate the progress of treatment, daily control of weight, blood pressure, heart rate, steps, and sleep duration, as well as the weekly control of fasting blood sugar (FBS), were advised.

**Figure 1:**
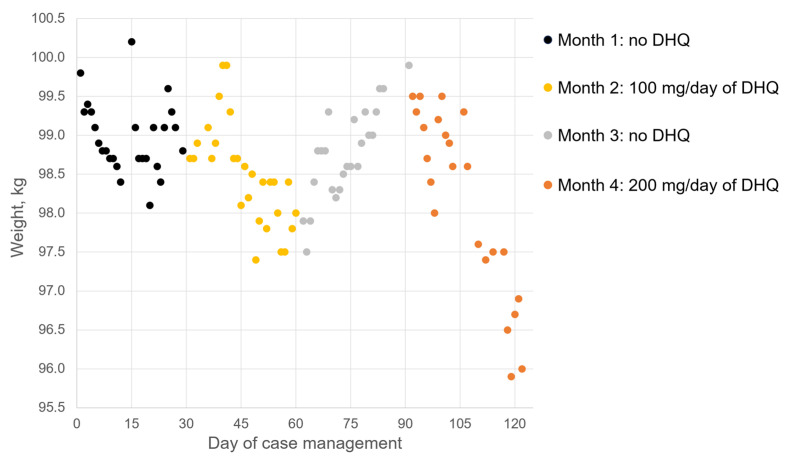
Dynamics of weight change during case management.

Over the next month, the patient demonstrated a high degree of compliance with the healthcare professional’s recommendations, and measurement data for 26 days were provided. The calculated dietary energy supply before the second visit was about 2100 kcal/day. Physical activity included weekly swimming (one h) and walking (approximately 8566 steps/day). The severity of walking-induced dyspnea decreased. The dynamics of weight change during Month 1 are reflected in Figure 1 as black dots. During this period, weight varied from 98.1 to 100.2 kg. There was a weak trend toward a decrease in body weight of −0.07 kg/week (*r* = 0.1949). The patient was not satisfied with his progress and expressed a desire to start the treatment with GLP-1RAs of Russian origin [21]. However, there were no indications for the use of these medicaments (such as type 2 diabetes mellites). Taking into account potential risks of GLP-1RA use [22,23,24], another method was suggested. Based on data in the literature regarding biological activity and a high safety profile [25], a dietary supplement based on DHQ was recommended at a dose of 100 mg/day.

Over the next month, the data from 27 days were collected. No significant difference was observed in dietary energy supply (2100 kcal/day) or physical activity (weekly swimming and walking, with approximately 8303 steps/day, were continued). During Month 2, weight varied from 97.8 to 99.9 kg. In this period, the rate of weight loss was estimated at 0.36 kg/week (see Figure 1, yellow dots) with a moderate time correlation (*r* = 0.6739). This result is closer to the recommended intensity of change for this parameter (0.5–1.0 kg/week). Walking-induced dyspnea was not reported. However, during intense physiological activity, the patient quickly felt fatigued (after 10–15 min of swimming), which had not been observed previously. The patient experienced dizziness once. In accordance with the recommendation of supplement use, the next month was DHQ-free.

Surprisingly, during Month 3, a weight increase was observed 0.44 kg/week (*r* = 0.8117) and the parameter value varied from 97.5 to 99.9 kg (see Figure 1, grey dots). In this period, the data were collected over 24 days. Weekly swimming and walking (approximately 8846 steps/day) were continued, as well as the diet with a calorie deficit. Fatigue during physiological activity was not reported.

During the next month, the recommended dose of DHQ was increased to 200 mg/day (100 mg twice a day). In this period, data were collected over 23 days. The trend toward weight loss continued: the parameter varied from 99.5 to 95.9 kg (see Figure 1, orange dots) and the mean rate of decrease was 0.73 kg/week (*r* = 0.8965). The patient followed the diet with a calorie deficit but did not visit the swimming pool. Walking intensity was estimated at approximately 8491 steps/day.

**Figure 2:**
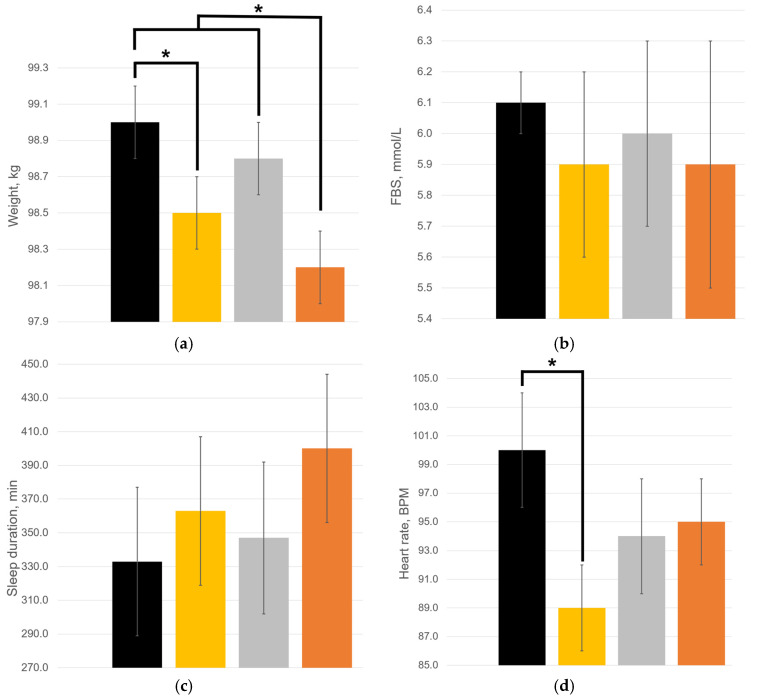
Dynamics of mean physiological data changes: (**a**) weight; (**b**) FBS; (**c**) sleep duration; (**d**) heart rate. Symbol * indicates significant difference between groups, *p* < 0.05. Black is for Month 1, no DHQ intake; yellow is for Month 2, 100 mg/day of DHQ; grey is for Month 3, no DHQ intake; orange is for Month 4, 200 mg/day of DHQ.

The mean values of the patient’s weight (Figure 2a) in the DHQ-free months did not differ significantly (*p* = 0.1871): 99.0 ± 0.2 kg and 98.8 ± 0.2 kg during Month 1 and Month 3, respectively. During treatment with 100 mg/day of DHQ (Month 2), the mean weight decreased to 98.5 ± 0.2 kg. A significant difference was observed compared to Month 1 (*p* = 0.0017) but not compared to Month 3 (*p* = 0.0688). The lowest mean weight was observed in Month 4 during treatment with 200 mg/day: it was 98.2 ± 0.2 kg, and the difference was significant compared to the DHQ-free months (*p* = 0.0326). In general, a very strong inverse correlation was observed between the DHQ dose and mean weight (*r* = −0.9699), as well as between the dose and the rate of weight change (*r* = 0.9334). Surprisingly, the correlations between these parameters and daily walking were lower: *r* = 0.4807 for mean weight and *r* = 0.6995 for the rate of weight change, respectively.

Although a very strong inverse correlation (*r* = −0.8182) was observed between the dose of DHQ and the FBS (Figure 2b), there was no significant difference in the mean values of this parameter (*p* = 0.4649). The mean FBS values were 6.1 ± 0.1, 5.9 ± 0.3, 6.0 ± 0.3, and 5.9 ± 0.4 mmol/L during Month 1, Month 2, Month 3, and Month 4, respectively. Additionally, a very strong association was found between the DHQ dose and sleep duration (*r* = 0.9730), as reflected in Figure 2c. Furthermore, sleep duration exhibited strong inverse correlations with mean weight (*r* = −0.8401), the rate of weight change (*r* = −0.9814), and the FBS (*r* = −0.8344).

**Figure 3:**
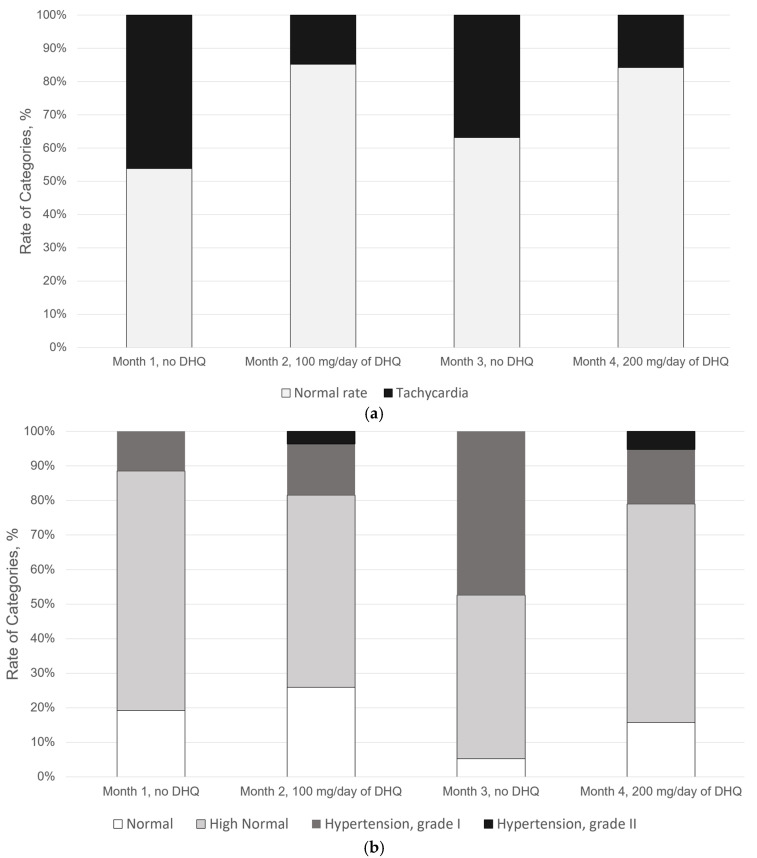
Dynamics of physiological state rates: (**a**) heart rate; (**b**) blood pressure.

As presented in Figure 2d, the start of DHQ treatment was associated with a significant decrease in the mean heart rate from 100 ± 4 BPM in Month 1 to 89 ± 3 BPM in Month 2 (*p* = 0.0001). During the next two months, the mean heart rate did not change significantly (*p* = 0.1653): 94 ± 4 BPM and 95 ± 5 BPM in Month 3 and Month 4, respectively. According to Figure 3a, DHQ intake was associated with a 3.1-fold reduction in tachycardia incidence, i.e., when the heart rate met tachycardia criteria (relative risk, RR = 0.32 [0.12, 0.88], *p* = 0.0130). Furthermore, Figure 3b shows that DHQ treatment interruption resulted in a 2.7-fold increase in hypertension incidence (RR = 2.7 [1.08, 6.75], *p* = 0.0254).

To suggest possible molecular mechanisms underlying the clinical observations, in silico analysis was performed.

### 2.2. Molecular Modeling

**Figure 4:**
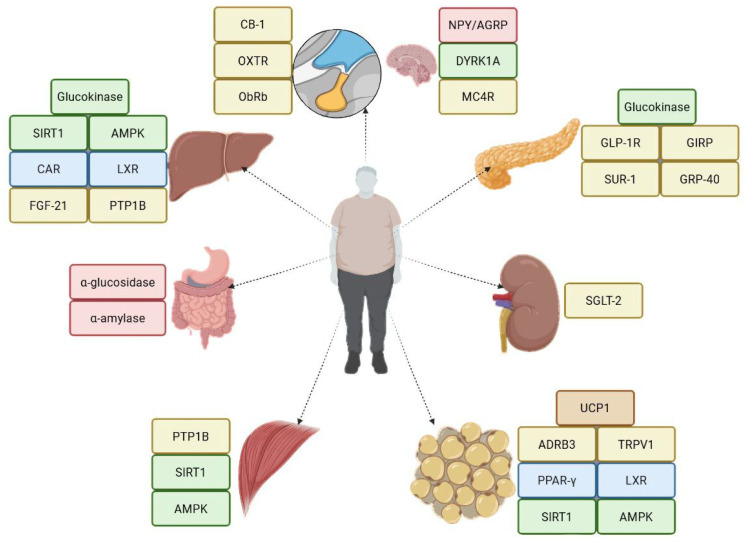
Localization of biological targets, implicated in body weight regulation.

The first step in the molecular modeling stage was the selection of potential biological targets for DHQ. Numerous receptors, localized in various tissues, can influence body weight (Figure 4). Based on the observed clinical findings, we focused on two key targets: β_3_-adrenoceptor (ADRB3) and peroxisome proliferator-activated receptor γ (PPARG). Activation of ADRB3 leads to a decrease in heart rate, initiates lipolysis, and reduces urine output. Agonists of PPARG increase insulin sensitivity, decrease gluconeogenesis and reduce susceptibility to angiotensin II, while also providing neuroprotective effects.

At the next stage, optimal 3D strictures of the biological targets were selected from the Protein Data Bank (PDB). The selection criteria were designed to minimize the potential risk of bias [26] and included the following characteristics: resolution (≤2.5 Å), organism (*Homo sapiens*), and ligand (the biological action resulted in weight loss). The summarized information for the selected models is presented in Table 1. Some other biological targets that suited these inclusion criteria, but showed inappropriate results of docking or redocking, are presented in Table S1.

**Figure 5:**
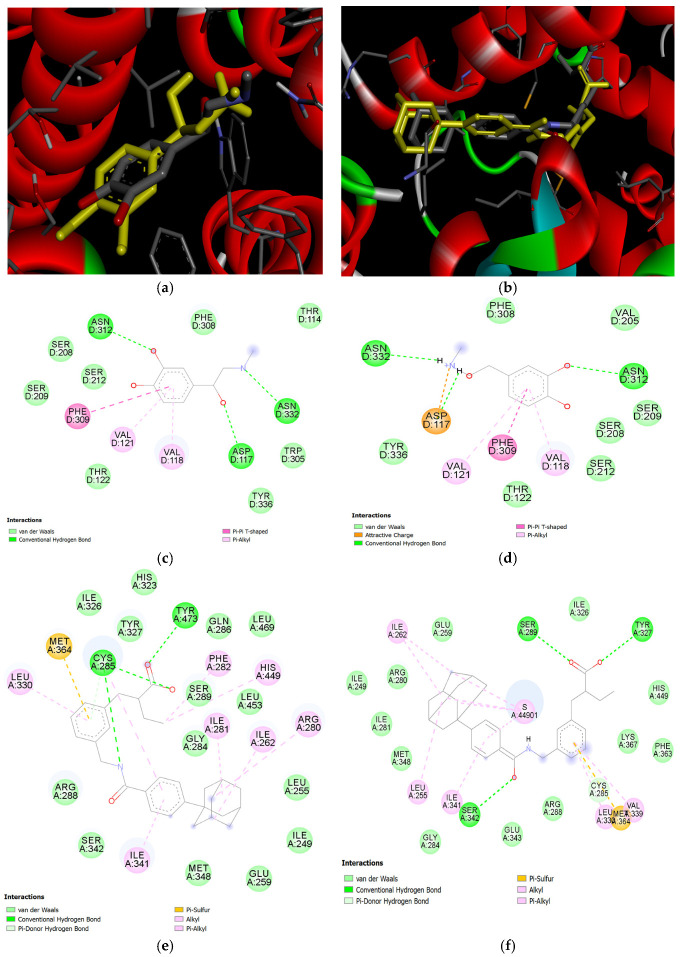
Results of redocking: (**a**) 3D structure of initial (grey) and redocked (yellow) ligand for ADRB3 in superposition; (**b**) 3D structure of initial (grey) and redocked (yellow) ligand for PPARG in superposition; (**c**) 2D diagram of intermolecular interactions of initial ligand for ADRB3; (**d**) 2D diagram of intermolecular interactions of redocked ligand for ADRB3; (**e**) 2D diagram of intermolecular interactions of initial ligand for PPARG; (**f**) 2D diagram of intermolecular interactions of redocked ligand for PPARG.

To validate the molecular docking methods, redocking experiments were performed using the initial ligands: epinephrine for ADBR3 and the synthetic agonist TIPP703 for PPARG. In both cases, the best binding affinities were lower then −4.777 kcal/mol, indicating the formation of stable intermolecular complexes between ligands and receptors (see Table 1). The positions of ligand molecules in the active sights did not differ significantly after several calculations (RMSD ≤ 2.0). Figure 5a,b confirm that redocked molecules bind to biological targets in the positions close to the initial ligands and interact with similar amino acid residues. For epinephrin—both in its initial and redocked position—the molecules form hydrogen and ionic bonds with asparagine 312, asparagine 332, and aspartate 117 (Figure 5c,d). Also, there is Pi–Pi stacking with phenylalanine 309, and it establishes lipophilic interactions with valine 118 and valine 121. A high similarity in interaction profiles is also observed for TIPP703 (Figure 5e,f). In both the initial ligand and the redocked structure, lipophilic interactions are present with isoleucine 262, leucine 330, and isoleucine 341. Furthermore, a Pi–sulfur interaction with methionine 364 is observed in both intermolecular complexes. Taken together, these findings support the relevance and reliability of the applied molecular modeling methods.

**Figure 6:**
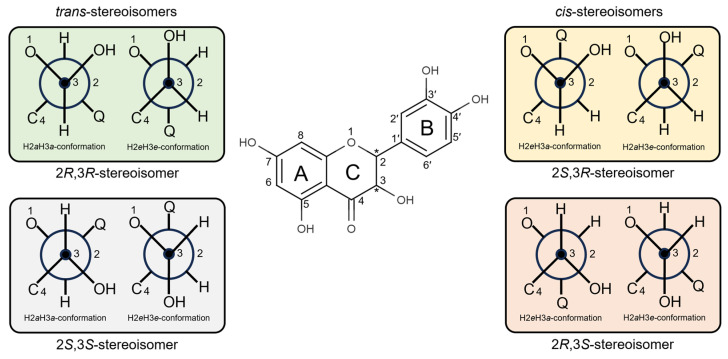
The structure of DHQ and possible conformations (presented in Newman projection formula) for different stereoisomers. The symbol * shows the position of stereocenters; Q = 3,4-dihydroxiphenyl.

The structure of DHQ contains two stereocenters at positions 2 and 3 (Figure 6), enabling the existence of all four stereoisomers of this flavonoid in both *trans*- and *cis*-configurations. Since the C-ring geometry is not planar, each stereoisomer can adopt one of two possible conformations depending on the orientation of the hydrogen atoms at the stereocenters: equatorial (*e*) or axial (*a*). Furthermore, at pH value 7.4, approximately 61.92% of DHQ molecules exhibit ionization of the OH group at position 7. Consequently, docking simulations were performed for both ionized and non-ionized forms of the molecule. In total, 144 calculations were conducted for each biological target.

**Figure 7:**
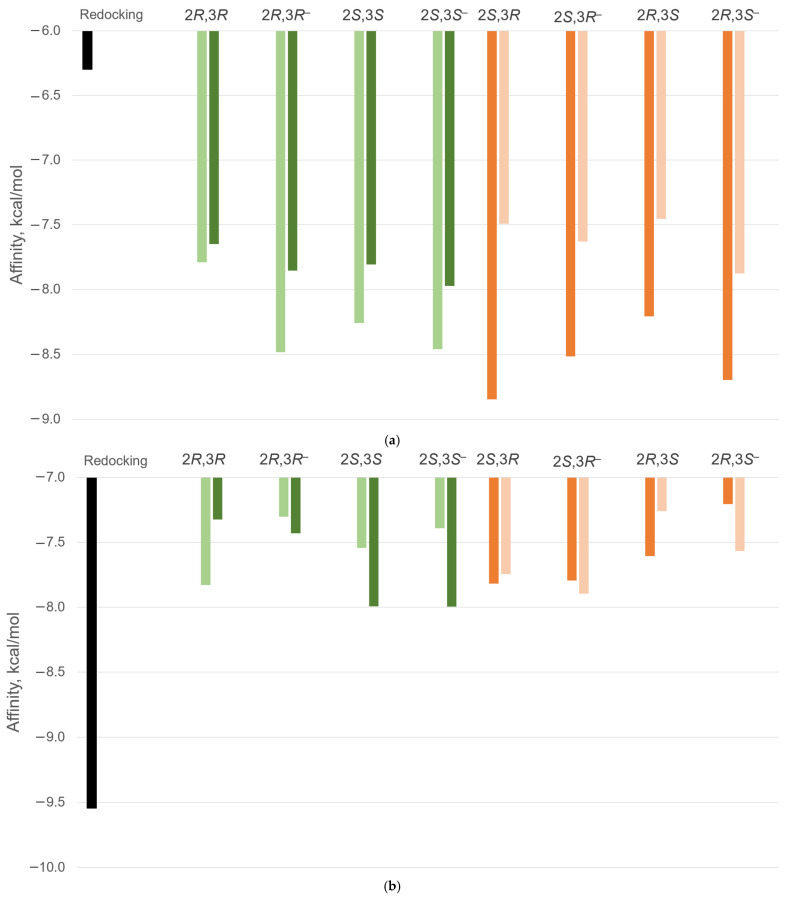
Dynamics of the mean physiological data changes: (**a**) ADRB3; (**b**) PPARG. Symbols—show DHQ ionization. Light green is for H2*e*H3*e*-conformation; dark green is for H2*a*H3*a*-conformation; dark orange is for H2*a*H3*e*-conformation; light orange is for H2*e*H3*a*-conformation.

The differences in binding affinities of DHQ stereoisomers for ADRB3 and PPARG are highlighted in Figure 7.

In ADRB3, the binding affinity of non- ionized *trans*-diastereomers varied from –7.649 kcal/mol (H2*a*H3*a*-conformation of 2*R*,3*R*-DHQ) to −8.258 kcal/mol (H2*e*H3*e*-conformation of the 2*S*,3*S*-isomer). For ionized *trans*-diastereomers, affinities were higher, ranging from −7.853 kcal/mol (H2*a*H3*a*-conformation of 2*R*,3*R*-DHQ) to −8.482 kcal/mol (H2*e*H3*e*-conformation of the same isomer). The *cis*-configuration was more preferable for forming an intermolecular complex with ADBR3. Among all analyzed structures, the highest affinity (−8.846 kcal/mol) was observed for the non-ionized H2*a*H3*e*-conformation of 2*S*,3*R*-DHQ. At the same time, the lowest affinity (−7.454 kcal/mol) was detected for the non-ionized H2*e*H3*a*-conformation of the 2*S*,3*R*-isomer. Notably, the data revealed an unexpected outcome: the difference in binding affinities to ADBR3 between conformers within *cis*-diastereomers (up to 1.392 kcal/mol) exceeds that observed for *trans*-isomers (up to 0.629 kcal/mol). Nevertheless, all DHQ structures exhibited higher affinity than the initial ligand.

Regarding the docking results with PPARG, TIPP703 exhibits higher affinity than any DHQ structure. Among the flavonoid’s stereoisomers, the highest affinity was observed for both ionized and non-ionized H2*a*H3*a*-conformation of 2*S*,3*S*-DHQ (−7.995 kcal/mol), whereas the lowest outcome was detected for the H2*a*H3*e*-conformation of ionized 2*R*,3*S*-DHQ (−7.206 kcal/mol). In general, molecules with 2*S*-configuration demonstrate 0.332 kcal/mol higher affinity compared to 2*R*-stereoisomers. The influence of the stereocenter configuration at position 3 was less pronounced (0.072 kcal/mol).

**Figure 8:**
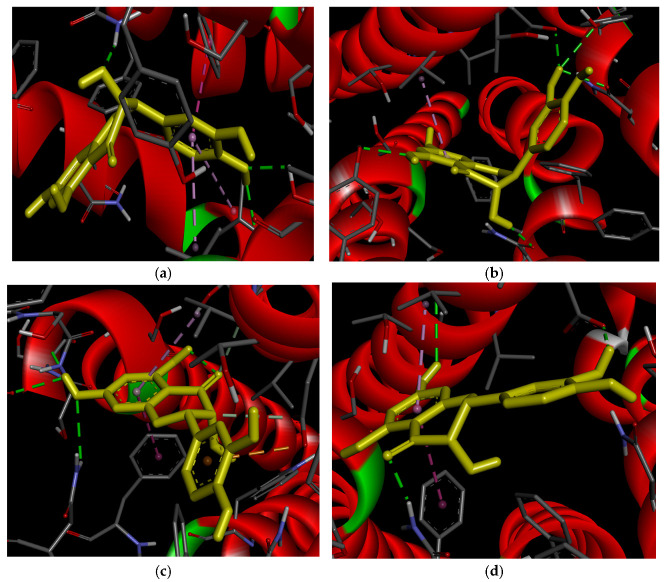
Results of docking of DHQ stereoisomers (yellow) with ADBR3: (**a**) 2*R*,3*R*-isomer; (**b**) 2*S*,3*S*-isomer; (**c**) 2*S*,3*R*-isomer; (**d**) 2*R*,3*S*-isomer. Green dashed lines reflect intermolecular hydrogen bonds; light green dashed lines reflect intermolecular van der Waals bonds; purple dashed lines reflect intermolecular Pi–Pi stacking; light purple dashed lines reflect intermolecular Pi–alkyl interactions; purple dashed lines reflect intermolecular Pi–anion interactions.

Figure 8 illustrates the interactions between DHQ stereoisomers and ADRB3. 2*R*,3*R*-DHQ forms hydrogen bonds with asparagine 312 and valine 118 via hydroxy groups at positions 3 and 4′ (Figure 8a). Additionally, ring B participates in lipophilic interactions with valine 118 and Pi–Pi stacking with phenylaniline 309. However, in this stereoisomer the benzopyran ring is orientated outside the receptor’s active site, so its functional groups do not engage in intermolecular bonds. The lowest RMSD value for 2*R*,3*R*-DHQ was recorded for the non-ionized H2*a*H3*a*-conformation (2.334), while the highest was observed for its ionized form (3.251).

The 2*S*,3*S*-isomer exhibits more extensive intermolecular interactions (Figure 8b). Hydrogen bonds form between hydroxy groups at positions 3 and 5 and the residues aspartate 117, asparagine 312, and asparagine 332. Lipophilic interactions also occur between ring A and valine 118. The generated complexes demonstrate good reproducibility, with RMSD values ranging from 2.112 to 2.773 across conformations.

Nevertheless, the 2*S*,3*R*-DHQ stereoisomer displays the most intense intermolecular interactions (Figure 8c), which correlates with its highest affinity. For this ligand, the following bonds were found: hydrogen bonds between the hydroxy group at position 7 and asparagine 312, the carbonyl group at position 4 and valine 118, and the hydrogen at position 3 and aspartate 117.

Additionally, Pi–Pi stacking occurs between ring A and phenylalanine 309, a Pi–anion interaction forms between ring B and aspartate 117, and lipophilic interactions are observed between ring A and valine 118. For this group, the highest RMSD values were detected for non-ionized H2*a*H3*e*- (2.905) and H2*e*H3*a*- (2.779) conformations.

The 2*S*,3*R*-isomer shows an interaction profile similar to 2*R*,3*R*-DHQ (Figure 8d). Hydroxy groups at positions 7 and 3′ form hydrogen bounds with valine 118 and aspartate 117, respectively, while the carbonyl group at position 4 interacts similarly with asparagine 312. Ring A participates in Pi–Pi stacking with phenylalanine 309 and lipophilic interactions with valine 118. However, the polar hydroxy group at position 3 is orientated toward a region rich in lipophilic amino acid residues. RMSD values for this group range from 2.544 (H2*a*H3*e*-conformation) to 3.005 (H2*e*H3*a*-conformation).

**Figure 9:**
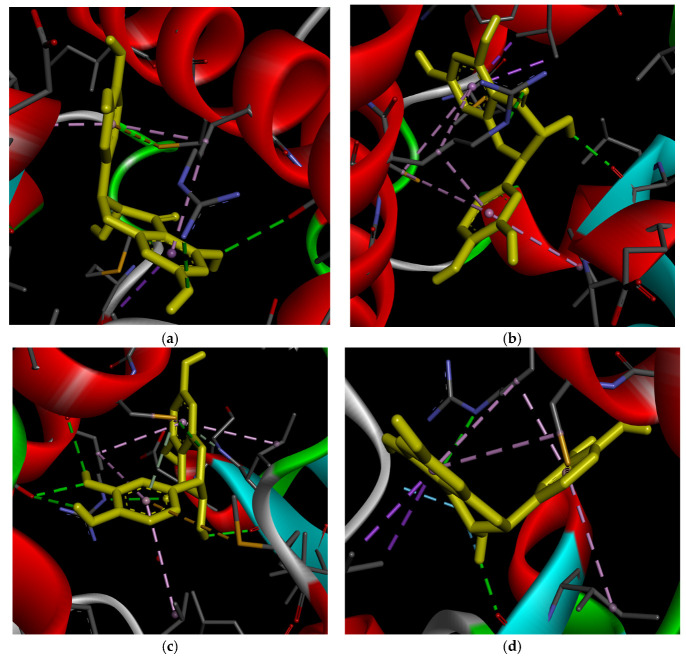
Results of DHQ stereoisomers (yellow) docking with PPARG: (**a**) 2*R*,3*R*-isomer; (**b**) 2*S*,3*S*-isomer; (**c**) 2*S*,3*R*-isomer; (**d**) 2*R*,3*S*-isomer. Green dashed lines reflect intermolecular hydrogen bonds; purple dashed lines reflect intermolecular Pi–sigma interactions (hyperconjugation); light purple dashed lines reflect intermolecular Pi–alkyl interactions; brown dashed lines reflect intermolecular Pi–sulfur interactions.

Figure 9 provides insights into the interactions between DHQ stereoisomers and PPARG. Overall, the observed profiles of intermolecular bonding profiles are similar to those from docking with ADBR3. The 2*R*,3*R*-, 2*S*,3*S*-, and 2*R*,3*S*-DHQ stereoisomers exhibit lower affinity. These compounds participate in lipophilic interactions with leucine 330 (via ring A) and isoleucine 341 (via ring B) and forming Pi–sulfur bonding with cysteine 285 (via ring B). In contrast, a more favorable interaction profile was observed for 2*S*,3*R*-DHQ: lipophilic interactions with leucine 330 (ring B), isoleucine 341 (ring A), and cysteine (ring A); a Pi–sulfur bond with cysteine 285 (ring B); and hydrogen bonds involving the oxygen atom of the benzopyran ring and cysteine residues. RMSD values for complexes with PPARG are higher compared to ADRB3 models: for 2*R*,3*R*-, 2*S*,3*S*-, 2*S*,3*R*-, and 2*R*,3*S*-stereoisomers, they were 9.916 (H2*a*H3*a*-conformation), 5.676 (H2*e*H3*e*-conformation), 9.099 (H2*a*H3*e*-conformation), and 9.829 (H2*a*H3*e*-conformation), respectively.

**Figure 10:**
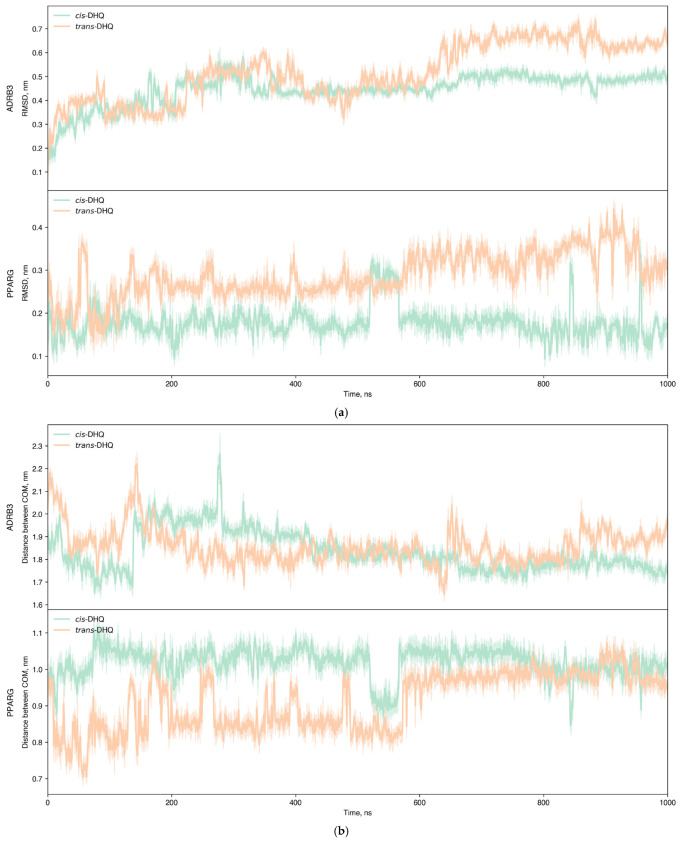
Shifts of *trans*-DHQ (orange) and *cis*-DHQ (green) in complexes with ADRB3 and PPARG during molecular dynamics simulation: (**a**) RMSD plots; (**b**) distance between COM plots.

An additional step in computational research on intermolecular interactions, which validates molecular docking results, is molecular dynamics analysis. This approach evaluates the stability of the generated complexes over time. For this analysis, the most representative diastereomer configurations were selected: 2*R*,3*R*-isomer as *trans*-DHQ and 2*S*,3*R*-isomer as *cis*-DHQ. Figure 10 illustrates the changes in the position of atoms (nm) as a function of simulation time (ns). At the RMSD plots (Figure 10a) the area under the curve for the *trans*-diastereomer exceeded that of the *cis*-isomer by 23.02% and 81.33% in ADRB3 and PPARG, respectively, indicating lower stability of the *trans*-configuration. In all cases, the RMSD value was lower than 2 Å for 1 μs of simulation: *trans*-DHQ exceeded values 0.7 nm and 0.4 nm in ADRB3 and PPARG, while for the *cis*-isomer it was 0.6 nm and 0.3 nm, respectively. Furthermore, distances between centers of mass (COMs) were calculated during molecular dynamics simulations (Figure 10b). In PPARG, both DHQ diastereomers showed low values of this parameter (<15 Å), reflecting the stability of the intermolecular complex, while in ADRB3 this parameter was higher.

**Figure 11:**
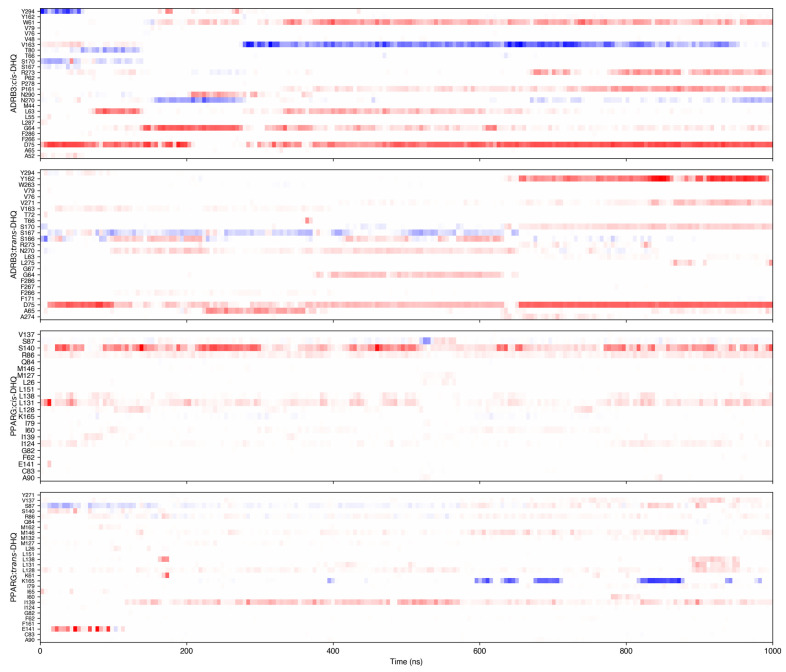
H-bonds maps for complex between DHQ diastereomers and ADRB3 and PPARG. Red reflects bonds, where DHQ is donor, and blue reflects bonds, where DHQ is acceptor.

At the same time, the intermolecular complex between ADRB3 and DHQ diastereomers was characterized by a higher number of H-bonds (Figure 11): during 1 μs of molecular dynamic simulation, the *trans*-isomer more frequently formed H-bonds with tryptophan 61, glycine 64, aspartate 75, serine 166, serine 167, serine 170, asparagine 270, and valine 271. At the same time, the *cis*-diastereomer formed H-bonds with ADRB-3 via the following amino acid residues: tryptophan 61, glycine 64, aspartate 75, leucine 63, valine 163, proline 161, asparagine 270, and arginine 273. In some moments, the number of H-bonds with ADRB3 reached 6 and 5 for *trans*- and *cis*-DHQ, respectively. At the same time, the PPARG *trans*-isomer formed H-bonds with serine 87, arginine 86, lysine 165, and isoleucine 139, while *cis*-DHQ was characterized by a high affinity to serine 87, arginine 86, leucine 131, and serine 140. In some points in time, the number of H-bonds with PPARG reached 6 and 5 for *trans*- and *cis*-DHQ, respectively. However, in the case of both biological targets, the H-bonds with 2*S*,3*R*-DHQ were more stable.

**Figure 12:**
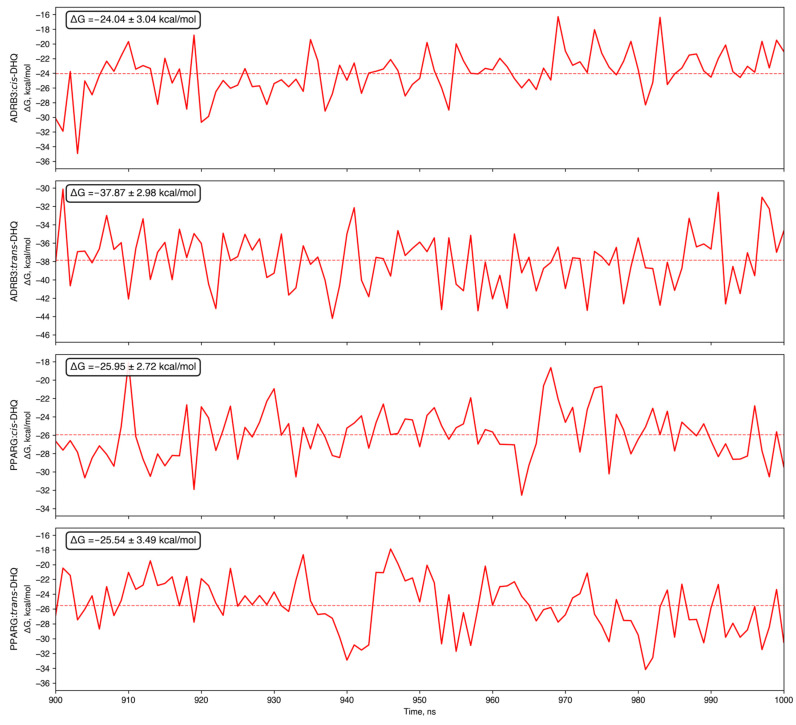
Binding free energy calculated using MM-PBSA approach of protein-ligand complexes during last 100 ns of molecular dynamics simulations. Dashed lines show the mean biding free energy.

According to binding free energy calculation data, intermolecular complexes were thermodynamically possible and stable throughout the simulation (Figure 12).

Taken together, the results of in silico research confirm the possibility of DHQ interactions with ADRB3 and PPARG.

## 3. Discussion

The present study aims to determine the molecular mechanisms underlying the anti-obesity effects of DHQ.

Molecular modeling methods provide a better understanding of biochemical processes responsible for observed biological effects. The methods of computational chemistry have been developing continuously [27,28,29]. When rigorously designed, in silico research can yield profound insights. For instance, such approaches have been used to explain interactions between the amyloid precursor protein and its local lipid environment [30], to shed light on the behavior of bovine serum albumin on atomically flat substrates [31], to design optimized artificial luciferases [32], and so on. The quality of computational analysis depends on its design. In the current research, human models of biological targets were selected at high resolution, ensuring that ligands in the active sites exhibited the required biological activity. The temperature and pH value of the medium were accounted for in the simulation. The validity of the computational approaches was confirmed through redocking experiments, which demonstrated good reproducibility of experimental results. Docking results were further validated by molecular dynamics simulations and found to be consistent with clinical case data. So, to minimize potential bias in molecular modeling, a complex of methods was employed.

Although commercially available active pharmaceutical substances are presented by a *trans*-diastereomer in 2*R*,3*R*-configuration [33], evidence from the literature indicates the ability of flavonoids to undergo epimerization in water medium [34,35,36]. DHQ diastereomers exhibit distinct pharmacokinetic profiles and pharmacological activities [37], which justified the inclusion of all DHQ enantiomers in the computational assessment. NMR data further confirm that flavonoid molecules can adopt multiple conformations [38]. Since the stability of intermolecular complexes can vary significantly depending on the conformer [39,40], each enantiomer was modeled in two conformations corresponding to local energy minima. This approach underlines the necessity of performing multiple calculations for each biological target.

The molecular docking results confirm that all analyzed DHQ models can form intermolecular complexes with biological targets. In all cases, the binding affinity was below –20 kJ/mol. Summarizing the data, we conclude that lipophilic interactions play a major role in ligand binding for both ADRB3 and PPARG, because there was not a significant affinity difference between ionized and non-ionized DHQ forms. Regarding chirality center configurations, *cis*-diastereomers showed a tendency towards higher affinity in ADRB3. This observation is supported by molecular dynamics data: The intermolecular system containing 2*S*,3*R*-DHQ exhibited a higher number of H-bonds and higher stability compared to models with 2*R*,3*R*-enantiomer. In PPARG, the differences in affinity between DHQ diastereomers were less pronounced. However, the 2*S*-configuration in this biological target was associated with lower energy states.

The interaction of DHQ with ADRB3 and PPARG is supported by clinical case data. The expected activation of ADRB3 may lead to a reduction in heart rate [41]. In the presented case, a significant 3.1-fold decrease in tachycardia frequency was observed during DHQ intake (*p* = 0.0130). On the other hand, suggested interruptions in PPARG may elevate blood pressure via regulation of the renin–angiotensin–aldosterone system [42]. In the reported case, interruptions in DHQ intake correlated with a 2.7-fold increase in hypertension cases (*p* = 0.0254). Also, in the observed individual, the DHQ dosage showed correlations with mean FBS levels (*r* = −0.8182) and body weight reduction (*r* = −0.9699). Additionally, the presence of a hypoglycemic effect is supported by the cases of fatigue during intense physiological activity in association with DHQ intake.

Previously, the anti-obesity effect of DHQ was noted by Hattori et al. it in a retrospective longitudinal study [43]. The authors attributed it to the activation of brown adipose tissue, where ADRB3 is localized. So, our outcomes of the clinical case and molecular modeling findings align with the evidence from previous observations. The hypoglycemic effects of DHQ have also been reported, though to the best of our knowledge all exclusively in animal models [17,35,44]. Additionally, the potential interaction between DHQ and PPARG was suggested by Kazazis et al. in 2014 [45].

Traditionally, flavonoids are described as antioxidants, with their biological effects attributed to free radical scavenging activities [46,47,48]. Since obesity is associated with oxidative stress [49], the use of natural polyphenols may benefit affected individuals. The antioxidant properties of these compounds may account for observed changes in the cardiovascular system status [50,51,52] and sleep quality improvement [53]. While mechanisms linking antioxidant actions to body weight reduction have been suggested [54,55], these do not exclude the possibility of DHQ interacting with specific biological targets. Furthermore, the delayed anti-obesity effect observed in Figure 1 may be explained by the low bioavailability of DHQ and the lag time required to achieve therapeutic blood concentrations. This delay is compounded by DHQ bonding with hemoglobin [56].

It is somewhat surprising that no anti-obesity effect of DHQ was reported before 2022 [57]. However, significant body weight reduction—reaching −25.41% in rats—was observed after 4 weeks of treatment with a daily oral dose of a 50 mg/kg of *cis*-isomer-enriched DHQ sample [58]. Although such substantial weight loss might be identified as a severe side effect, DHQ is recognized for its high safety profile [59,60,61]. Furthermore, when a DHQ-based remedy was registered in Russia, no effects on body weight were noted [62]. There are several possible explanations for this fact. Firstly, the therapeutic effect may manifest only under pathological conditions, which were not the focus of earlier research. Secondly, increasing the single dose from 40 to 100 mg likely resulted in higher exposure to *cis*-DHQ due to epimerization: initial DHQ studies primarily used the *trans*-diastereomer [63], whereas current findings highlight the greater activity of the *cis*-isomer.

The present study has several limitations. The generalizability of the findings is constrained by the single-case design. The observed effects may appear due to the placebo effect, as well as host-related factors. Also, as the monitoring was carried out at home, many parameters (such as biochemical parameters or amount of extracted urine) slip under the radar of investigators, as they can only be controlled in a hospital. Additionally, both ADRB3 and PPARG affect the amount of excreted urine, so the impact of this factor should be assessed in future research. Furthermore, polyphenols are characterized by high multiplicity of affinity to diverse biological targets. This makes it harder to explain the precise molecular mechanisms underlying the observed effects. Specifically, a confounding influence from alternative signaling pathways cannot be ruled out. Therefore, larger studies employing enzyme assays, relevant animal models, and larger numbers of patients are needed.

Despite these limitations, this study highlights a promising way for anti-obesity therapy, demonstrating efficacy in real-world clinical practice. The molecular modeling results are validated by the robust design of the computational study and the observations of the clinical case. Thus, the insights gained from the presented data will inform the design of future experiments aimed at developing novel anti-obesity treatment based on natural flavonoids with particular emphasis on *cis*-isomer-enriched formulations.

## 4. Materials and Methods

### 4.1. Clinical Case

The collected data were used after the patient had signed an informed consent form.

DHQ (Lavitol^®^, Ametis JSC, Blagoveshchensk, Russia) was administrated as a dietary supplement at a dose of 100–200 mg/day. The data collected one month before the treatment were used as a control, and all data were completed. To minimize the potential risk of bias, early outcome events were out of focus of the researchers. Additionally, the outcome assessors were unaware of the intervention received.

The patient collected his clinical data by himself using home medical equipment. Body weight was measured using the Mi Smart Scale 2 (Anhui Huami lnformation Technology Co., Hefei, China) and recorded in the Zepp Life app (v. 6.15.0, Anhui Huami lnformation Technology Co., Hefei, China). Data on heart rate, blood pressure, and FBS were stored in the Umniy Monitoring Zdorovia app (v. 1.70.004, Doverie LLC, Moscow, Russia), which received inputs from the Smart Blood Pressure Monitor (SberZdorovie, Moscow, Russia) and the Contour Plus One (Ascensia Diabetes Care, Basel, Switzerland). The number of steps and sleep duration were tracked via the Mi Smart Band 6 (Anhui Huami lnformation Technology Co., Hefei, China) using the Mi Fitness app (v. 3.47.1i, Beijing Xiaomi Co., Beijing, China).

### 4.2. Molecular Docking

The generation of DHQ models, conformational search, and ionization assessment (at 300 K) were performed using MarvinBeans (v. 5.2.4, ChemAxon Ltd., Budapest, Hungary) [64]. The structures were optimized in Avogadro (v. 1.2, Avogadro Chemistry, Pittsburg, PA, USA) [65] using the MMFF94 force field [66].

The protein structures of ADRB3 [67] and PPARG [68] were obtained from the RCSB Protein Data Bank. Structure processing included removing any ligands, ions, co-factors, and water molecules that might interfere with docking, as well as the assignment of hydrogen atoms to the protein model. Missing residues were constructed using SwissModel (Computational Structural Biology Group, Basel, Switzerland) [69].

Molecular docking was performed using Webina (v. 1.0.5, Durrant Lab, Pittsburg, PA, USA) [70]. System validation was also carried out in the same software. All calculations were repeated 9 times, and the resulting affinities and RMSD data were reported. BIOVIA Discovery Studio Visualizer (v. 4.5, Dassault Systèmes, Vélizy-Villacoublay, France) [71] was used to visually assess the molecular docking results and generate 2D diagrams of intermolecular interactions.

### 4.3. Molecular Dynamics

Proteins investigated in this study are listed in Table 1. Conformations with the best scoring were used. The ADRB3 receptor was embedded into an explicitly defined lipid bilayer composed of POPC (1-palmitoyl-2-oleoyl-sn-glycero-3-phosphocholine) lipids. The system was constructed using the CHARMM-GUI web service [72,73] (Membrane Builder module) with approximate lateral dimensions of the lipid bilayer of 8.5 × 8.5 nm. The PPARG protein was placed in a cubic box with a minimum distance of 1 nm between the protein and the box edges and was solvated using the Solution Builder module. Protonation states of amino acid residues were assigned according to pH 7.4. The PPM 2.0 web server [74] was used to predict the positioning of ADRB3 in the membrane. Molecular dynamics simulations were performed using GROMACS (version 2024.4) [75] with the CHARMM36m force field [76]. DHQ parameters were developed using CGenFF (CHARMM General Force Field) [77]. Water molecules were described using the TIP3P model [78].

Initial atomic velocities were assigned according to a Maxwell–Boltzmann distribution at 311.15 K, and bond lengths were constrained using the LINCS algorithm [79]. A 1.2 nm cutoff was applied for Lennard-Jones interactions, with dispersion corrections included in the calculations of both energy and pressure. Electrostatic interactions were computed using the particle-mesh Ewald (PME) method with a grid spacing of 0.12 nm and a real-space cutoff of 1.2 nm. Production molecular dynamics simulations were carried out for 1 μs with an integration time step of 2 fs in the NPT ensemble. Single molecular dynamics runs were performed with independently generated initial velocities. Prior to production simulations, the systems were subjected to energy minimization followed by equilibration molecular dynamics simulations, with gradual release of positional restraints on atoms according to the standard CHARMM-GUI protocol. Temperature and pressure were maintained using the V-rescale thermostat [80] and the C-rescale barostat (if required) [81].

Trajectory analysis was performed using built-in GROMACS modules as well as the MDAnalysis package [82]. Binding free energies were estimated using the MM-PBSA (Molecular Mechanics Poisson–Boltzmann Surface Area) method implemented in gmxMMPBSA software (version 1.6.4) [83,84].

### 4.4. Statistical Analysis

Continuous data from the case report were presented as the mean ± half-width of the confidence interval (α = 0.05). To assess the significance of differences between data collected in different months, a one-way analysis of variance (ANOVA) was performed. *p*-values < 0.05 were considered to be significant.

Categorical data from the case report were summarized as frequencies. To evaluate the significance of observed differences in binary data, relative risks were calculated and confidence intervals were reported (α = 0.05). *p*-values < 0.05 were considered to be significant.

## 5. Conclusions

This case adds to the growing body of evidence suggesting that DHQ may be a safe and effective treatment for obesity. In the presented clinical case, a body weight reduction rate of 0.73 kg/week during DHQ administration in a daily dose of 200 mg was observed, with no severe side effects in a patient with obesity and prediabetes. A second major finding is that the observed biological effects can be explained by DHQ’s interaction with ADRB3 and PPARG. To the best of our knowledge, this is the first study to report on the impact of DHQ configuration on its affinity to these biological targets and to discuss its clinical relevance for body weight control. The insights gained from this study may contribute to our understanding of flavonoids not merely as antioxidants but also as active ingredients that selectively interact with receptors. The methods developed for DHQ could be applied to other flavonoids. If future investigations confirm these results, they may serve as a foundation for developing a new class of anti-obesity remedies that act via ADRB3.

## Figures and Tables

**Table 1:** Confirmation of models of selected biological targets.

Biological Target			Characteristics of Selected 3D Model from PDB					Results of Redocking	
Name	Localization	pH Value	PDB ID	Organism	Method	Resolution, Å	Initial Ligand	Affinity, kcal/mol	RMSD
ADRB3	Adipose tissue	7.4	9IJE	*Homo* *sapiens*	Electron microscopy	2.34	Epinephrine	−6.297	1.297
PPARG	Adipose tissue	7.4	2ZNO	*Homo* *sapiens*	X-Ray diffraction	2.40	Synthetic agonist TIPP703	−9.545	1.615

## Data Availability

The original contributions presented in this study are included in the article and Supplementary Materials. Further inquiries can be directed to the corresponding author.

## Supplementary Materials

The following supporting information can be downloaded at: https://www.mdpi.com/article/10.3390/ijms27062846/s1.

## Abbreviations

The following abbreviations are used in this manuscript:

ADRB3	β3-adrenoceptor
ANOVA	analysis of variance
COM	center of mass
GLP-1RAs	glucagon-like peptide-1 receptor agonists
DHQ	dihydroquercetin
FBS	fasting blood sugar
PDB	Protein Data Bank
PPARG	peroxisome proliferator-activated receptor γ
RR	relative risk
