# Supplementary material for: Dihydroquercetin in Obesity and Prediabetes: Case Report and Insights from Molecular Modeling
Source: Int J Mol Sci. 2026 Mar 20;27(6):2846. doi: 10.3390/ijms27062846 (PMC13026499; doi:10.3390/ijms27062846)
Supplement: Supplementary file 1 [file ijms-27-02846-s001.zip › ijms-4173481-supplementary.pdf]

# Dihydroquercetin in Obesity and Prediabetes: Case Report and Insights from Molecular Modeling

**Roman P. Terekhov[1,*], Amir Taldaev[2,3], Artem A. Svotin[1], Denis I. Pankov[1], Evgenia M. Sukhova[1], David A. Manukov[1], Ketelina Bergel[1], Maria D. Korochkina[1] and Irina A. Selivanova[1]**

[1] Nelyubin Institute of Pharmacy, Sechenov First Moscow State Medical University, 119991 Moscow, Russia; terekhov_r_p@staff.sechenov.ru
[2] Laboratory for the Study of Single Biomacromolecules, Institute of Biomedical Chemistry, 119121 Moscow, Russia; t-amir@bk.ru
[3] Research Center for Molecular Mechanisms of Aging and Age-Related Diseases, Moscow Center for Advanced Studies, 123592 Moscow, Russia; t-amir@bk.ru
* Correspondence: terekhov_r_p@staff.sechenov.ru; Tel.: +7 (499) 749-79-91

# Supplementary Materials

**Table S1.** Results of redocking and DHQ docking of unselected biological targets.

| **Biological target** | | **Results of redocking** | | **Affinity of DHQ stereoisomers, kcal/mol** | | | |
|---|---|---|---|---|---|---|---|
| **Name** | **PDB ID** | **Affinity, kcal/mol** | **RMSD** | **2*R*,3*R*** | **2*S*,3*S*** | **2*S*,3*R*** | **2*R*,3*S*** |
| DYRK1A | 6LN1 | –6.154 | 2.342 | –1.583 | –3.241 | –3.385 | –3.186 |
| PTP1B | 1XBO | –5.080 | 3.323 | –3.280 | –3.241 | –2.321 | –3.301 |
| SUR1 | 6JB3 | –6.848 | 6.722 | –6.595 | –6.727 | –6.775 | –6.743 |
| Glucokinase | 3VF6 | –7.351 | 5.104 | –6.411 | –6.004 | –5.915 | –6.173 |
